# Developmental Alterations in the Diffusion Tensor Imaging Analysis Along the Perivascular Space Index Suggest Possible Glymphatic-Related Mechanisms Underlying Excitation/Inhibition Imbalance and Psychosis Vulnerability in 22q11.2 Deletion Syndrome

**DOI:** 10.1016/j.bpsgos.2026.100713

**Published:** 2026-02-27

**Authors:** Alessandro Pascucci, Silas Forrer, Corrado Sandini, Valentina Mancini, Yasser Alemán-Gómez, Stephan Eliez, Farnaz Delavari

**Affiliations:** a Developmental Imaging and Psychopathology Laboratory, Department of Psychiatry, University of Geneva School of Medicine, Geneva, Switzerland; b Synapsy Centre for Neuroscience and Mental Health Research, University of Geneva, Geneva, Switzerland; c Medical Image Processing Laboratory, Neuro-X Institute, École Polytechnique Fédérale de Lausanne, Lausanne, Switzerland; d MRC Brain Network Dynamics Unit and Oxford Centre for Integrative Neuroimaging, Nuffield Department of Clinical Neurosciences, University of Oxford, Oxford, United Kingdom; e Department of Psychiatry, University of Oxford, Oxford, United Kingdom; f Radiology Department, Lausanne University Hospital and University of Lausanne, Lausanne, Switzerland; g Department of Genetic Medicine and Development, University of Geneva School of Medicine, Geneva, Switzerland

**Keywords:** 22q11.2 deletion syndrome, Diffusion tensor imaging (DTI), Excitation/inhibition balance, Glymphatic system, Magnetic resonance spectroscopy, Psychosis

## Abstract

**Background:**

Impairment of the glymphatic system may contribute to atypical brain development and increased vulnerability to psychiatric conditions such as psychosis. In particular, disrupted glymphatic efficiency may affect neurochemical homeostasis during critical maturational windows, leading to structural and circuit-level alterations. However, its role in early neurodevelopmental trajectories remains largely unexplored.

**Methods:**

We combined longitudinal diffusion tensor imaging (DTI) in 85 individuals with 22q11.2 deletion syndrome (22q11DS), a neurodevelopmental condition associated with elevated psychosis risk (143 scans), with cross-sectional magnetic resonance spectroscopy in a subset of 39 individuals with 22q11DS. Glymphatic function was estimated indirectly using the DTI analysis along the perivascular space (ALPS) index, a diffusion-based proxy derived from manual and automated region of interest placement. Excitation/inhibition ratio was assessed in the right hippocampus via cerebrospinal fluid–corrected combined glutamate-glutamine signal (Glx) and GABA (gamma-aminobutyric acid) levels.

**Results:**

The ALPS index was significantly reduced in individuals with 22q11DS compared with control participants (*p* = .017), especially in the right hemisphere. Individuals with positive psychotic symptoms (PPS+) showed a divergent developmental trajectory, failing to exhibit the age-related ALPS increase seen in PPS− individuals (group × age interaction: *p* = .009). In a subset with spectroscopy data (*n* = 39), lower ALPS index predicted higher Glx/GABA ratio in the right hippocampus (*p* = .002).

**Conclusions:**

These findings provide in vivo evidence that glymphatic-related dysfunction, as indexed by the DTI-ALPS proxy, emerges early and follows atypical developmental trajectories in individuals at risk for psychosis. An impaired ALPS index is also associated with excitatory/inhibitory imbalance. This dysfunction may represent a novel pathway contributing to psychosis vulnerability and a potential target for early intervention.

## Plain Language Summary

Individuals with 22q11.2 deletion syndrome are at high risk for psychosis, yet early brain mechanisms remain unclear. Using MRI, we examined the ALPS index, a diffusion-based proxy for perivascular fluid–related brain clearance. ALPS index values were reduced early in life and showed abnormal developmental trajectories in individuals who later developed psychotic symptoms. Lower ALPS index values were also associated with hippocampal excitatory/inhibitory imbalance, linking early brain clearance alterations to psychosis vulnerability.

Understanding how deviations in normal brain development result in psychiatric illness requires an integrative framework encompassing genetic vulnerabilities, environmental exposures, and the biological systems that mediate their interaction. Among these systems, the glymphatic clearance network has emerged as a promising point of convergence, potentially linking diverse risk factors to disrupted brain homeostasis during critical periods of development. This perivascular network, known as the glymphatic system (GS), drives cerebrospinal fluid (CSF) into brain parenchyma through astrocytic AQP4 channels and directs interstitial by-products, including potassium, excess glutamate, cytokines, and misfolded proteins, toward venous and meningeal lymphatic pathways (1, 2, 3, 4). Efficient glymphatic flow is essential for synaptic remodeling, metabolite recycling, and overall circuit maturation (5). Its disruption can amplify excitotoxicity, oxidative stress, and neuroinflammatory processes that have been repeatedly implicated in psychosis pathophysiology (6, 7, 8). Thus, glymphatic dysfunction may represent a mechanistic pathway linking disrupted neurophysiology to psychosis, consistent with postmortem, CSF, and positron emission tomography evidence of neuroinflammation in schizophrenia and high-risk states (9, 10, 11, 12, 13, 14).

Among the downstream consequences of impaired glymphatic flow, altered excitation/inhibition (E/I) balance represents a key mechanism underlying psychosis vulnerability. E/I balance, governed by glutamatergic and GABAergic (gamma-aminobutyric acidergic) signaling, is essential for synaptic refinement and circuit stability (15, 16, 17) and is disrupted both in psychosis (18) and in 22q11.2 deletion syndrome (22q11DS), where reduced GABAergic tone and excessive glutamatergic activity have been linked to hippocampal atrophy and increased psychosis risk (19). Given the central role of the hippocampus in psychosis-related circuits (20), impaired local clearance may facilitate glutamate accumulation and trigger excitotoxic cascades (21). Therefore, inefficient metabolite clearance represents an additional pathway contributing to circuit-level imbalance.

Human imaging studies using diffusion-based proxies have linked impaired glymphatic function to neurodegenerative, neurodevelopmental, and psychiatric conditions, mostly using cross-sectional designs (22, 23, 24, 25, 26, 27, 28, 29, 30). Importantly, the glymphatic network itself matures postnatally, with progressive organization of perivascular pathways throughout adolescence and into early adulthood (31, 32, 33). However, the developmental trajectories of glymphatic function and their interaction with mechanisms known to contribute to psychosis remain largely uncharacterized. Addressing this gap is critical, as enhancing glymphatic clearance during key developmental windows may represent a viable preventive opportunity (34, 35, 36).

22q11DS offers a valuable model for studying GS development. This microdeletion is associated with up to a 40-fold increased risk for schizophrenia and other neurodevelopmental disorders, making it a relevant clinical and biological model for psychiatric risk (37, 38, 39, 40). Several structural components of the GS appear to be directly affected in 22q11DS. Cellular and animal studies have reported compromised blood-brain barrier (BBB) integrity (41, 42, 43), altered astrocyte maturation (44,45), and impaired ependymal cilia function (46)—all crucial elements for efficient glymphatic clearance.

To estimate GS function in vivo, we leveraged the diffusion tensor imaging (DTI) analysis along the perivascular space (ALPS) index. This imaging approach provides a reproducible and noninvasive proxy for glymphatic clearance efficiency, previously validated in studies of aging and neurodegenerative disorders (47, 48, 49, 50).

In the largest longitudinal cohort of individuals with 22q11.2DS studied to date, we mapped age-related changes in the ALPS index and tested whether this proxy for impaired brain clearance was linked to the emergence of positive psychotic symptoms (PPSs). We also investigated a subgroup of deletion carriers with available magnetic resonance spectroscopy (MRS) datasets to examine the relationship between the ALPS index and E/I imbalance, a neurochemical alteration already implicated both in 22q11DS populations and in psychosis more broadly (19,51). Through this integrative approach, we aimed to determine whether and when alterations in the ALPS index first emerge during development, how they intersect with established pathogenic pathways, and where intervention efforts might best be targeted to support healthy brain maturation.

## Methods and Materials

### Participants

This study included longitudinal assessments of 85 individuals with a confirmed diagnosis of 22q11DS (143 scans; mean age = 18.92 ± 6.36 years; 42 female and 43 male) and 83 healthy control (HC) participants (115 scans; mean age = 16.28 ± 6.42 years; 43 female and 40 male), all recruited as part of the Swiss 22q11DS longitudinal cohort. At scan, participants were ages 5 to 35 years and contributed a total of 258 DTI scans across multiple time points. Demographic and clinical characteristics of the sample are provided in Table 1. Details of magnetic resonance imaging (MRI) data acquisition and processing are provided in Supplemental Methods.

**Table 1:** Participant Characteristics

Demographics, Per Scan					
Group	Number of Scans	Number of Participants, Total, Female/Male	Age at Scan, Years	FSIQ	Average Years Between Visits
HC	115	83, 43/40	16.3 (6.4)	NA	5.05 (1.88)
22q11DS	143	85, 42/43	18.9 (6.4)	71.6 (11.5)	5.30 (2.11)
PPS+	68	38, 19/19	19.4 (6.5)	70.9 (11.7)	5.23 (1.88)
PPS−	68	40, 20/20	19.5 (5.8)	72.2 (11.3)	5.38 (2.33)
PPS indeterminate	7	7, 3/4	9.2 (2.1)	NA	NA

Clinical Characteristics, 22q11DS Only, Per Scan					
	PPS+		PPS−		*p* Value
ADHD	49 (72.1%)		46 (67.6%)		.709
Anxiety Disorders	53 (77.9%)		44 (64.7%)		.129
Mood Disorders	41 (60.3%)		30 (44.1%)		.086
Neurodevelopmental Disorders	49 (72.1%)		46 (67.6%)		.709
Antipsychotics	30 (44.1%)		6 (8.8%)		<.001
Antidepressants	41 (60.3%)		25 (36.8%)		.010
Mood Stabilizers	6 (8.8%)		0 (0.0%)		.028
Stimulants	27 (39.7%)		39 (57.4%)		.059
Benzodiazepines	9 (13.2%)		4 (5.9%)		.243
Medicated	49 (72.1%)		48 (70.6%)		>.99
≥1 Psychiatric Diagnosis	64 (94.1%)		58 (85.3%)		.156

Values are presented as *n*, *n* (%), or mean (SD).

*p* Values refer to comparisons between groups (PPS+ vs. PPS−) performed using χ^2^ or Fisher’s exact tests for categorical variables. IQ was measured using WISC-III for children and WAIS-III for adults. Years between visits represent the mean intervisit interval per participant, averaged across participants with ≥2 scans.

22q11DS, 22q11.2 deletion syndrome; ADHD, attention-deficit/hyperactivity disorder; FSIQ, Full Scale IQ; HC, healthy control; NA, not available; PPS, positive psychotic symptom; WAIS-III, Wechsler Adult Intelligence Scale, Third Edition; WISC-III, Wechsler Intelligence Scale for Children, Third Edition.

HC participants were recruited from siblings (38 of 85 HC participants [44.7%] belonged to families that also included a participant with 22q11.2DS) of deletion carriers and through an open call from the Geneva state school system. They were screened to exclude any current or past neurological or psychiatric diagnoses, learning disabilities, history of prematurity, or use of psychotropic medications. In the 22q11DS group, the microdeletion was confirmed using quantitative fluorescent polymerase chain reaction. Written informed consent was obtained from all participants and/or their legal guardians. The study protocol was approved by the Institutional Review Board of Geneva University School of Medicine and conducted in accordance with the Declaration of Helsinki.

### Clinical Assessment

All participants with 22q11DS underwent clinical evaluation by an expert psychiatrist (SE), including a semistructured interview and the Structured Interview for Psychosis-Risk Syndromes (SIPS). Consistent with previous publications from the same group, the presence of clinically significant PPSs was defined as a score ≥3 on any of the 5 positive symptom items (P1–P5) at any time points. This criterion was chosen as it has been verified to be one of the criteria for ultra-high-risk status in this population (52, 53, 54, 55, 56).

### Data Acquisition

All participants underwent MRI on a 3T Siemens scanner (Trio and Prisma-Fit) using standardized acquisition protocols. Diffusion-weighted images were acquired with 30 directions (b = 1000 s/mm^2^), and MEGA-PRESS (Mescher-Garwood point resolved spectroscopy) proton MRS (^1^H-MRS) was used to quantify GABA+ and combined glutamate-glutamine signal (Glx) in the right hippocampus. Voxel placement was guided by individual T1-weighted scans. For full acquisition parameters and preprocessing details, see Supplemental Methods.

### ALPS Index Method

The ALPS index was computed using an established methodology as a diffusion-based proxy for perivascular diffusivity (47,48,57,58). Diffusion tensor components along the 3 orthogonal axes (diffusivity in the right-left direction [Dxx]; diffusivity in the anterior-posterior direction [Dyy]; diffusivity in the inferior-superior direction [Dzz]) were extracted in native space (Supplemental Methods). Bilateral spherical regions of interest (ROIs) were placed at the level of the lateral ventricles in projection and association white matter. ROIs were positioned adjacent to medullary veins, which course along the x-axis, presumed to align with perivascular spaces, while avoiding areas of fiber crossing in the same direction (49). The ALPS index was calculated per hemisphere as

(1) $$\text{ALPS}\;\text{index}=\frac{\text{mean}\;\left(\text{Dx}\_\text{proj},\text{Dx}\_\text{assoc}\right)}{\text{mean}\;\left(\text{Dy}\_\text{proj},\text{Dz}\_\text{assoc}\right)}$$

Dx_proj and Dx_assoc indicate diffusivity parallel to the presumed perivascular orientation within projection and association fiber ROIs, respectively, whereas Dy_proj and Dz_assoc reflect diffusivity orthogonal to that orientation (see Supplemental Methods).

A bilateral ALPS value was obtained by averaging hemispheres. Findings were replicated using an automated atlas-based ROI pipeline, and a corpus callosum (CC) Dxx negative-control analysis was performed at the same axial level using the same mixed-effects framework (Supplemental Methods).

### Statistical Analysis

Left and right ALPS indices were computed separately, and outliers were removed using the IQR (±1.5) method, applied independently by group. Bilateral mean ALPS values were derived from scans with valid indices in both hemispheres. Psychosis-stratified analyses included 78 individuals with 22q11DS with valid SIPS assessments. Based on positive symptom scores (P1–P5 ≥ 3), 38 were classified as psychosis positive (PPS+), and 40 as psychosis negative (PPS−), contributing 123 scans. Analyses accounted for the nested longitudinal structure of the data, with detailed scan counts and exclusions reported in Supplemental Methods. Linear mixed-effects models were used to evaluate group differences and developmental trajectories. Models included fixed effects for diagnostic group, age (mean centered), sex, and the group × age interaction, along with a participant-level random intercept and random slope for age to account for repeated measures. The same modeling approach was applied to ALPS indices derived from both manual and automated ROI methods. Statistical significance was set at *p* < .05.

Multiple comparisons were controlled for using false discovery rate (FDR) correction, and sensitivity analyses examining the influence of Full Scale IQ were performed within the 22q11DS group; full details and results are provided in the Supplement.

### MRS Processing and Analysis

In a subset of 39 individuals with 22q11DS (mean age = 21.6 ± 6.9 years; 25 male), both high-quality spectroscopy and diffusion data were available. ^1^H-MRS data were processed using Gannet, a widely used toolbox for GABA-edited MRS analysis, to quantify GABA+, Glx, and unsuppressed water signals and to compute Glx/GABA ratios as a proxy for E/I balance (11,19,59,60).

Tissue segmentation from T1-weighted images enabled correction for partial volume and modeling of tissue-related relaxation properties. Data quality was assessed, and spectra exceeding predefined fit error thresholds were excluded. Further methodological details, including modeling parameters and quality control thresholds, are provided in Supplemental Methods.

Linear regressions were performed in Python using the statsmodels package (OLS function) to test the association between the ALPS index (predictor) and the Glx/GABA ratio (outcome) while controlling for age and sex. Analyses were conducted using left, right, and bilateral ALPS values, with outliers excluded via the IQR method (IQR ±1.5). A single outlier scan was excluded due to a low right ALPS value; no Glx/GABA outliers were detected. All data were cross-sectional (i.e., 1 scan per participant).

## Results

### Early ALPS-Measured Glymphatic Dysfunction in 22q11DS

**Figure 1:**
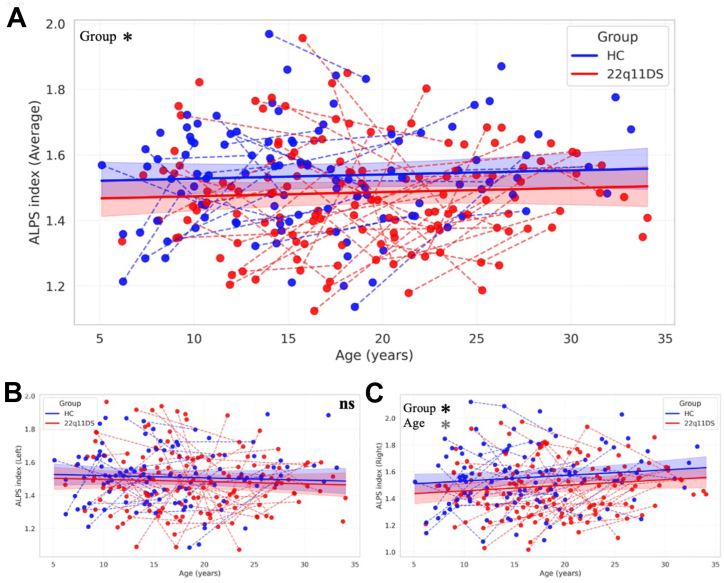
Age-related trajectories of ALPS index in individuals with 22q11.2DS and HC participants based on manual region of interest placement. **(A)** Average ALPS index plotted against age in individuals with 22q11DS (red) and HC participants (blue). Each dot represents a scan; dashed lines connect longitudinal scans from the same participant. Shaded areas represent 95% CIs of the fitted linear mixed-effects models. A significant group difference in the average ALPS index was observed, with lower values in the 22q11DS group (*p* = .017). **(B)** The ALPS index in the left hemisphere showed a nonsignificant trend toward lower values in individuals with 22q11DS compared with HC participants (*p* = .253). **(C)** The right hemisphere ALPS index demonstrated a significant reduction in the 22q11DS group (*p* = .022), alongside a significant effect of age (*p* = .037), indicating a developmental modulation specific to this region. All models included age (mean centered), group, sex, and age × group interaction as fixed effects and participant-level random slopes for age to account for repeated measures. Black asterisks indicate significant group differences, and gray asterisks indicate significant age effects. ∗*p* < .05. 22q11.2DS, 22q11.2 deletion syndrome; ALPS, analysis along the perivascular space; HC, healthy control; ns, not significant.

To evaluate group-level differences in the trajectory of glymphatic clearance efficiency, we compared ALPS index values in individuals with 22q11DS and HC participants over age. Mixed-effects models revealed a significant reduction in the bilateral average ALPS index in the 22q11DS group compared with the control group (β = −0.05; 95% CI, −0.10 to −0.01; *p* = .017) (Figure 1A and Table S4A). This effect was also significant in the right hemisphere (β = −0.07; 95% CI, −0.13 to −0.01; *p* = .022) (Figure 1C and Table S4C), confirming a right-lateralized decrease in the ALPS index. The left hemisphere showed a slightly reduced ALPS index in the 22q11DS group, but the difference did not reach statistical significance (β = −0.03; 95% CI, −0.08 to 0.02; *p* = .253) (Figure 1B and Table S4B).

The group × age interaction was not significant in any model, indicating parallel developmental trajectories across groups. Age was not a significant predictor in the left or bilateral ALPS models, suggesting stability of these measures across development. In contrast, age was a significant positive predictor of right ALPS values (β = 0.01; 95% CI, 0.00 to 0.01; *p* = .037) (Figure 1C and Table S4C), indicating a modest age-related increase in the right-hemisphere ALPS index consistent with physiological maturation. As the group × age interaction was not significant, this effect was observed in both individuals with 22q11DS and HC participants, suggesting that the right ALPS index undergoes a modest age-related modulation across development, consistent with physiological maturation rather than a group-specific pathological change. Sex did not emerge as a significant predictor in any model. The summary of all full models is displayed in Table S4. These findings provide convergent evidence that glymphatic clearance is already compromised in 22q11DS from early childhood (as young as 5 years, the lower bound of our cohort), particularly in the right hemisphere, potentially reflecting early structural or vascular anomalies reported in this population.

These effects were further confirmed using an automated ALPS index computation pipeline (see Supplemental Results), which algorithmically defined ROIs in each participant’s native space. The automated analysis replicated the group differences observed with manually defined ROIs, supporting the reliability of ALPS index measurements and reinforcing the robustness of our findings. Furthermore, a control analysis of Dxx extracted from bilateral CC ROIs placed at the same axial level as the ALPS ROIs showed a pattern opposite to that of the ALPS findings: Dxx was significantly increased in individuals with 22q11DS relative to HC participants (β = −4.82 × 10^−4^, *p* = −1.27 × 10^−11^) (Supplemental Results).

All main group effects remained significant after FDR correction (see Table S9). To account for potential familial nonindependence, we reestimated all models including a family-level random intercept. The right-hemisphere ALPS difference between individuals with 22q11.2DS and HC participants remained significant (β = −0.070, *p* = .020) (Table S10).

### ALPS-Measured Glymphatic Development in Relation to Psychosis Risk in 22q11DS

**Figure 2:**
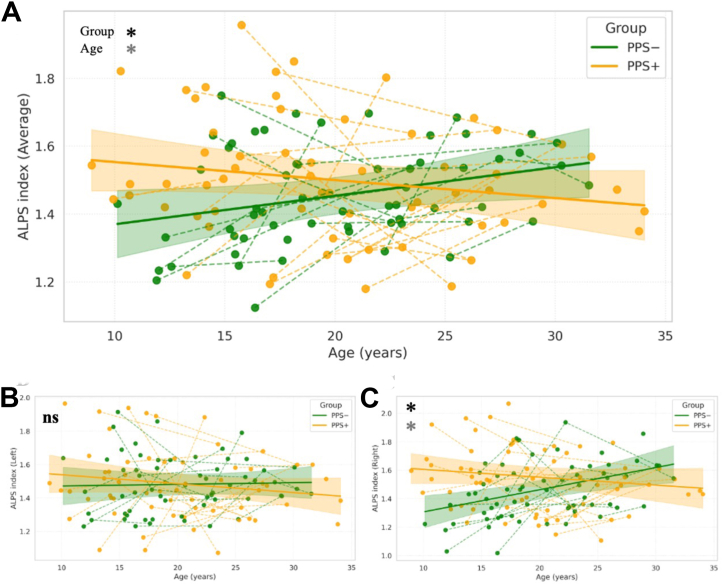
Divergent age-related trajectories of glymphatic efficiency in 22q11DS individuals with and without PPSs. **(A)** The average ALPS index plotted against age in individuals with 22q11.2DS stratified by presence (PPS+, orange) or absence (PPS−, green) of clinically significant PPSs. Each dot represents a scan; dashed lines connect longitudinal data from the same individual. Shaded areas represent 95% CIs of the fitted linear mixed-effects models. Although no significant group-level difference was observed at the mean age (*p* = .141), the group × age interaction was significant (*p* = .009), indicating divergent developmental trajectories; PPS− individuals exhibited increasing glymphatic efficiency with age, while PPS+ individuals showed flat or declining trajectories. **(B)** In the left hemisphere, no significant effects were detected for group (*p* = .955), age (*p* = .785), or their interaction (*p* = .292). **(C)** In the right hemisphere, both the main effect of group (*p* = .025) and the group × age interaction (*p* = .001) were significant. PPS+ individuals showed higher ALPS index values at the mean age compared with PPS− individuals but failed to exhibit the normative age-related increase in glymphatic efficiency, resulting in a flatter or declining developmental trajectory. All models included age (mean centered), group, sex, and age × group interaction as fixed effects and participant-level random slopes for age to account for repeated measures. Black asterisks indicate significant group differences, and gray asterisks indicate significant age effects. ∗*p* < .05. 22q11.2DS, 22q11.2 deletion syndrome; ALPS, analysis along the perivascular space; ns, not significant; PPS, positive psychotic symptom.

To evaluate whether ALPS index trajectories during development differ as a function of psychosis liability, we compared values across age between PPS+ and PPS− individuals. Mixed-effects models revealed no significant group-level difference in the ALPS index at the mean age in the bilateral average (*p* = .141) (Figure 2A) or left hemisphere (*p* = .955) (Figure 2B) models. However, in the right hemisphere, a significant main effect of group was observed (β = 0.09; 95% CI, 0.01 to 0.17; *p* = .025) (Figure 2C and Table S6C), with PPS+ individuals exhibiting higher ALPS index values than PPS− individuals at the mean age of the sample.

Crucially, significant group × age interactions were found in both the average and right hemisphere models (average: β = −0.01, 95% CI, −0.02 to −0.00, *p* = .009; right: β = −0.02, 95% CI, −0.03 to −0.01, *p* = .001) (Figure 2A, C and Table S6A, C), indicating divergent developmental trajectories between the 2 subgroups. While PPS− individuals showed the expected age-related increase in the ALPS index, PPS+ individuals showed a markedly blunted or declining trajectory, particularly in the right hemisphere. This pattern suggests that although PPS+ individuals exhibited elevated ALPS values in early life, these values did not follow the age-related increase observed in PPS− individuals.

Both interaction effects for the average and right ALPS indices survived FDR correction, whereas left-hemisphere effects remained nonsignificant (Table S9).

In the left hemisphere, no significant effects were detected for group (*p* = .955), age (*p* = .785), or their interaction (*p* = .292) (Figure 2B). Similarly, sex was not a significant predictor in any of the models (average: *p* = .805, right: *p* = .504, left: *p* = .987), indicating that the observed differences in glymphatic trajectories are unlikely to be attributable to sex.

Overall, these findings indicate altered developmental trajectories of the ALPS index in individuals with 22q11DS meeting clinical thresholds for PPSs, with effects being more pronounced in the right hemisphere. A full summary of model estimates and significance values is provided in Table S6.

### Sensitivity to Cognitive Functioning

Controlling for Full Scale IQ did not alter ALPS measures, PPS-related effects, or developmental trajectories; full results are reported in the Supplement (Table S8A–E).

### ALPS-Measured Glymphatic Function and E/I Imbalance in 22q11DS

**Figure 3:**
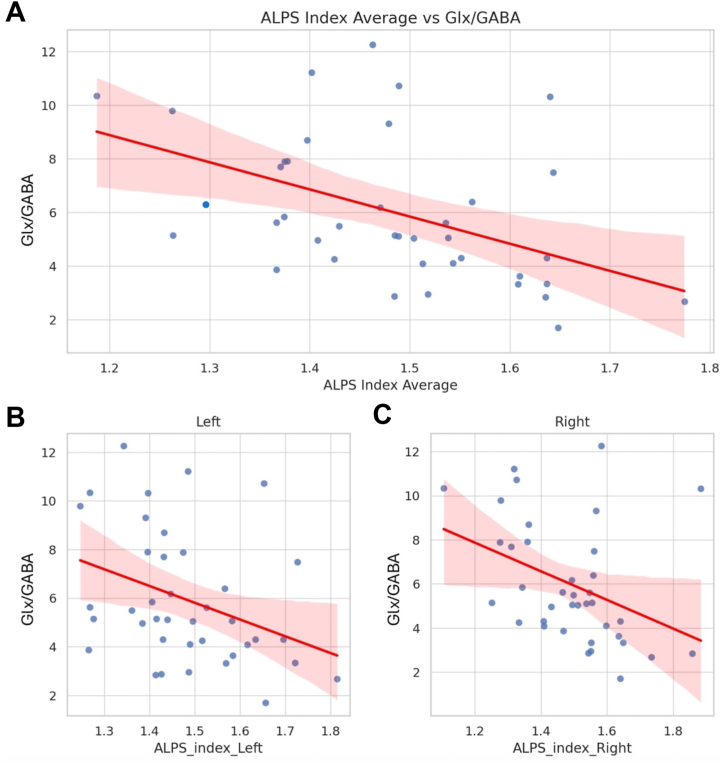
Association between the ALPS index and Glx/GABA ratio in 22q11 deletion syndrome. Scatterplots depict the linear association between the ALPS index and the Glx/GABA ratio (excitatory-inhibitory balance) in the right hippocampus for the bilateral average (top), left hemisphere (bottom left), and right hemisphere (bottom right) ALPS indices. Each dot represents a participant. Red regression lines represent the best-fit line from linear models, with shaded areas indicating the 95% CIs. All models controlled for age and sex. Significant negative associations were observed for all 3 ALPS metrics, with the strongest effect found for the average index (*p* = .0022). ALPS, analysis along the perivascular space; GABA, gamma-aminobutyric acid; Glx, combined glutamate-glutamine signal.

Linear regression analyses were performed on 38 scans for the right and average ALPS indices, and on 39 scans for the left ALPS index, after outlier removal, as detailed in the Methods and Materials section. A significant negative association emerged between the ALPS index and the Glx/GABA ratio in the right hippocampus, while controlling for age and sex. In particular, the average ALPS index significantly predicted the Glx/GABA ratio (β = −11.02, *p* = .0022), accounting for 25.5% of the variance (*R*^2^ = 0.255) (Figure 3 and Table S7).

When we analyzed hemispheres separately, the left ALPS index also significantly predicted the Glx/GABA ratio (β = −7.66, *p* = .0181, *R*^2^ = 0.159) (Figure 3 and Table S7), as did the right ALPS index (β = −6.77, *p* = .0148, *R*^2^ = 0.174) (Figure 3 and Table S7).

These findings indicate that a reduced ALPS index is robustly associated with increased E/I imbalance measured in our cohort in the right hippocampus in 22q11DS, with consistent effects across both hemispheres and the strongest association observed for the bilateral average index.

Full model results are reported in Table S7.

All ALPS-Glx/GABA associations remained statistically significant after FDR correction (Table S9).

## Discussion

### Developmental Alteration in the ALPS Index as a Potential Early Vulnerability Marker of Psychosis in 22q11DS

The ALPS index, derived from directional diffusivity along perivascular spaces, has been proposed as a noninvasive proxy for assessing glymphatic function (47). To our knowledge, this is the first study to characterize developmental alterations in this metric in the context of psychosis risk. We observed that individuals with 22q11DS already exhibited lower ALPS index values during childhood, potentially indicating early disruption in perivascular fluid dynamics. These findings may reflect the downstream effects of the 22q11.2 deletion on neurovascular (41) and astroglial (61) systems, both of which are implicated in the regulation of interstitial clearance (44,62).

One possible contributing factor is the hemizygous deletion of the gene encoding CLDN5, a tight-junction protein expressed in brain microvascular endothelial cells. Prior evidence from animal models suggests that CLDN5 haploinsufficiency may compromise tight-junction integrity and increase BBB permeability, potentially altering perivascular homeostasis (63, 64, 65). Increased BBB leakiness could, in turn, affect fluid exchange and directional diffusivity in perivascular spaces, thereby influencing the ALPS index.

Additionally, glymphatic transport is thought to depend on AQP4 water channels located in astrocytic endfeet (2,66). Several genes within the 22q11.2 locus are involved in mitochondrial-dependent processes underlying astrocyte development and function, and astrocytic abnormalities have been reported in animal models of 22q11DS (44,45). Impaired AQP4 localization or astrocytic polarity may interfere with CSF influx and restrict water diffusivity along perivascular conduits, possibly contributing to the reduced ALPS values observed. While speculative, the combination of BBB dysfunction and astrocytic alterations provides a plausible cellular basis for observing an early glymphatic inefficiency in 22q11DS in our results.

Taking advantage of the longitudinal design, we stratified individuals with 22q11DS according to the later emergence of clinically significant PPSs (PPS+ vs. PPS−). ALPS trajectories diverged between these groups early in life and prior to the onset of symptoms, suggesting that alterations in perivascular fluid dynamics may precede and potentially contribute to later vulnerability. Interestingly, at baseline, the PPS+ group showed slightly higher ALPS values than the PPS− group. However, over time, PPS− individuals displayed an age-related increase in the ALPS index, consistent with physiological glymphatic maturation, whereas PPS+ individuals failed to show this developmental progression.

Based on existing literature suggesting that the ALPS index captures aspects of periventricular diffusivity related to perivascular fluid dynamics (47,49), the transient early elevation that we observed in the PPS+ subgroup could represent a compensatory upregulation in response to early stressors or immune challenges. 22q11DS is associated with broad immune dysregulation, including T-cell deficiency and impaired mucosal immunity (67, 68, 69, 70). It is conceivable that only a subset of individuals experiences chronic or recurrent infections, resulting in sustained peripheral and central immune activation. This may elevate the brain’s metabolic and inflammatory load, particularly in perivascular compartments. In this context, an initial increase in ALPS values in PPS+ individuals may reflect a temporary adaptive response to such stressors. However, failure to maintain this compensation over time may signal the collapse of clearance capacity under increasing developmental strain, potentially contributing to downstream neurodevelopmental disruptions and psychosis onset.

### The ALPS Index and Hippocampal E/I Imbalance

Excitotoxicity has long been proposed as a contributor to psychosis pathophysiology (6,71, 72, 73). Excess extracellular glutamate, arising from impaired reuptake, NMDA receptor hypofunction, or altered cortical drive, can lead to oxidative stress, interneuron dysfunction, and structural atrophy (74). Prior studies have linked hippocampal glutamate abnormalities to prodromal symptoms and hippocampal volume loss in both idiopathic psychosis and 22q11DS (19,20,75).

In this study, we observed a significant inverse correlation between the ALPS index and the Glx/GABA ratio in the right hippocampus. While correlational, these findings are consistent with the hypothesis that alterations in perivascular diffusivity may interfere with glutamate clearance, allowing excitatory by-products to accumulate and contributing to local circuit hyperexcitability.

Notably, this association was observed specifically in the right hippocampus, a region known to exhibit high metabolic demand (76) and dense vascularization (77). The hippocampus is also particularly vulnerable to oxidative stress and neuroinflammation during early development (8,73), which may amplify its sensitivity to impaired metabolite clearance. Although hippocampal Glx/GABA ratios may reflect global neurochemical alterations, it is possible that the hippocampus functions as an early sentinel region. Future studies using whole-brain MRS and multimodal imaging will be essential to determine whether this association generalizes beyond the hippocampus.

**Figure 4:**
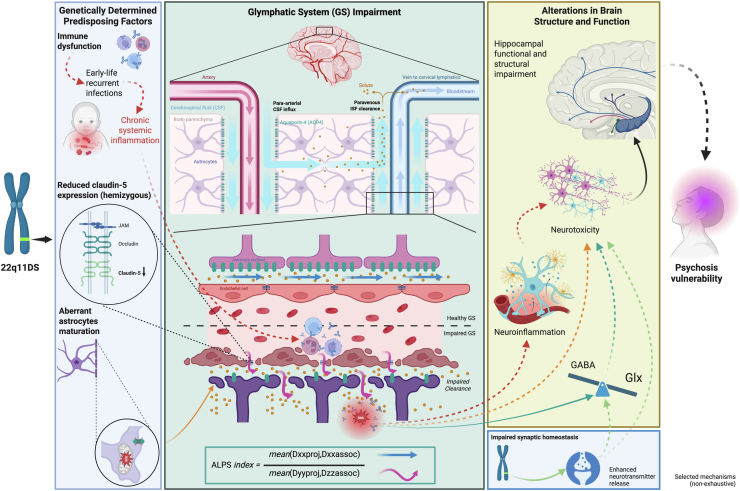
Proposed mechanistic framework linking 22q11.2DS to glymphatic dysfunction and psychosis vulnerability. The hemizygous 22q11.2 deletion confers several genetically determined predispositions, including systemic immune dysfunction due to impaired maturation of T lymphocytes and impaired mucosal immunity, leading to increased susceptibility to early-life infections and recurrent systemic inflammation. Additional vulnerabilities include reduced claudin-5 expression, which compromises blood-brain barrier integrity, and aberrant astrocytic maturation associated with haploinsufficiency of mitochondrial-related genes within the deleted locus. Mitochondrial deficits increase oxidative stress and impair astrocytic maturation and polarization, thereby disrupting AQP4 localization at astrocytic endfeet. AQP4 channels are essential for cerebrospinal-interstitial fluid interchange, and their loss of polarity compromises the integrity of the external wall of perivascular spaces, a critical element of glymphatic function. These converging mechanisms are hypothesized to impair glymphatic system efficiency, leading to reduced perivascular clearance, with the ALPS index serving as a noninvasive proxy for this process. Impaired clearance may promote solute accumulation within the brain parenchyma, contributing to neuroinflammation, E/I imbalance (indexed by Glx/GABA ratios here), oxidative stress (reactive oxygen species increase), and subsequent neurotoxicity. In parallel, haploinsufficiency of presynaptic genes within the deleted locus affects synaptic development and plasticity, compromising homeostatic mechanisms and potentially leading to enhanced neurotransmitter release that might contribute to E/I imbalance and ultimately neurotoxicity. Due to their high metabolic demand, dense vascularization, and susceptibility to oxidative stress and excitotoxicity, hippocampal circuits are especially vulnerable and tend to be compromised early in development. Furthermore, evidence suggests that hippocampal dysfunction can disrupt dopaminergic circuitry, driving aberrant dopaminergic activity that is closely linked to the emergence of psychotic symptoms. The schematic highlights a nonexhaustive set of mechanisms converging on glymphatic dysfunction and illustrates their interactions across levels, from molecular deficits to clinical phenotype. ALPS index = mean (Dxxproj, Dxxassoc)/mean (Dyyproj, Dzzassoc). (Figure created in BioRender.) 22q11.2DS, 22q11.2 deletion syndrome; ALPS, analysis along the perivascular space; Dxxassoc, diffusivity along the x-axis in association fibers; Dxxproj, diffusivity along the x-axis in projection fibers; Dyyproj, diffusivity along the y-axis in projection fibers; Dzzassoc, diffusivity along the z-axis in association fibers; E/I, excitation/inhibition; GABA, gamma-aminobutyric acid; Glx, combined glutamate-glutamine signal; ISF, interstitial fluid.

Taken together, our findings support a developmental framework in which early cellular vulnerabilities in 22q11DS, potentially affecting astrocytic function and BBB integrity, may be associated with alterations in periventricular microstructural and clearance-related processes from childhood. These alterations appear to persist or worsen in individuals who go on to develop psychotic symptoms (PPS+) and are accompanied by hippocampal E/I imbalance. The convergence of impaired interstitial clearance and neurochemical dysregulation may amplify excitotoxic cascades and network instability, potentially contributing to psychosis risk. As illustrated in Figure 4, these findings can be conceptualized within a broader mechanistic framework linking the 22q11.2 deletion to increased psychosis risk.

While preliminary, this model suggests that ALPS-indexed perivascular diffusivity may represent a modifiable component of early neurodevelopmental trajectories. Consistent with this, early pharmacological and neuromodulatory studies in other clinical populations have reported increases in the ALPS index, associated with concurrent improvements in sleep and cognition (78, 79, 80).

### Interpretation of the ALPS Index: Periventricular Microstructure Versus Glymphatic-Related Transport

While the ALPS index was initially proposed as a diffusion-based proxy sensitive to water mobility along perivascular pathways (47), subsequent methodological work has clarified that it primarily reflects directional diffusivity in periventricular white matter and may be influenced by local microstructural characteristics (81), including fiber-crossing geometry (82), making it sensitive, but not specific, to glymphatic-related transport. This nuance is particularly relevant in 22q11.2DS, a condition characterized by widespread white matter alterations such as reduced diffusivity, reduced tract volume, and smaller or more densely packed axons (83,84). To assess whether ALPS index reductions simply reflected these canonical microstructural abnormalities, we performed a negative-control analysis in the CC. At the same axial level as the ALPS ROIs, callosal Dxx was significantly increased in 22q11.2DS, whereas the opposite was true for periventricular ALPS reductions (Figure S3B), arguing against global white matter confounds. Together with the robust association between ALPS values and hippocampal Glx/GABA ratios, these findings support a balanced interpretation whereby ALPS captures biologically meaningful periventricular diffusivity, with glymphatic-related processes remaining a plausible contributor.

### Limitations and Future Perspectives

Several limitations of the current study should be noted. First, following the standard published methodology for the calculation of the ALPS index (47), ROI placement was performed manually, which may have introduced operator bias. Therefore, we implemented an automated, atlas-based ALPS ROI approach that replicated the main findings.

Second, group differences in the ALPS index exhibited a right-lateralized pattern, with the most pronounced effects observed in the right hemisphere, although similar trends were present on the left. This hemispheric asymmetry is consistent with prior reports of right-lateralized structural and functional anomalies in 22q11DS (85,86) and may reflect increased vulnerability of the right hemisphere to neurodevelopmental perturbations. Notably, right hippocampal atrophy has been implicated in schizophrenia (87) and 22q11DS (88), with altered developmental trajectory of right hippocampal volume in PPS+ participants emerging on the right side, further supporting the relevance of this asymmetry for psychosis risk.

Third, in our dataset, spectroscopy measures were available only from the right hippocampus and were cross-sectional in nature. This limits the interpretation of ALPS-metabolite associations to a unilateral and noncausal level. In addition, this study represents a secondary analysis of an ongoing longitudinal 22q11DS cohort, with partial overlap of hippocampal MRS data with prior work (19). Future whole-brain, longitudinal spectroscopy studies will be needed to more comprehensively characterize metabolite dynamics.

Fourth, the current longitudinal cohort extends only into early adulthood, limiting our ability to assess whether ALPS-indexed alterations contribute to later-onset neurodegenerative outcomes in 22q11DS. Individuals with 22q11DS exhibit a markedly elevated risk for early-onset Parkinson’s disease (89), and longitudinal studies extending into later adulthood will be required to clarify this association.

Finally, translational efforts bridging human imaging with mechanistic animal work will be essential to clarify causal pathways. For example, 22q11DS mouse models, such as LgDel, have demonstrated mitochondrial deficits affecting astrocyte development (44,90) and may serve as valuable platforms to investigate how genetic haploinsufficiency perturbs glymphatic architecture and brain clearance function, ultimately impacting circuit dynamics relevant to psychosis. If alterations captured by the ALPS index contribute to a second-hit mechanism in 22q11DS, they may point to modifiable perivascular processes relevant for early interventions aimed at restoring physiological homeostasis rather than treating downstream symptoms.

## Supplementary Material

Supplemental Methods, Results, Figures S1–S3, and Tables S1–S10

Key Resources Table
