# Supplementary material for: Developmental Alterations in the Diffusion Tensor Imaging Analysis Along the Perivascular Space Index Suggest Possible Glymphatic-Related Mechanisms Underlying Excitation/Inhibition Imbalance and Psychosis Vulnerability in 22q11.2 Deletion Syndrome
Source: Biol Psychiatry Glob Open Sci. 2026 Feb 27;6(3):100713. doi: 10.1016/j.bpsgos.2026.100713 (PMC13091000; doi:10.1016/j.bpsgos.2026.100713)
Supplement: Supplemental Methods, Results, Figures S1–S3, and Tables S1–S10 [file mmc1.pdf]

SUPPLEMENTARY INFORMATION

Developmental Alterations in the Diffusion Tensor Imaging Along the Perivascular Space Index Suggest Possible Glymphatic-Related Mechanisms Underlying Excitation/Inhibition Imbalance and Psychosis Vulnerability in 22q11.2 Deletion Syndrome

Pascucci *et al.*

TABLE OF CONTENTS

1. SUPPLEMENTARY METHODS
2. SUPPLEMENTARY RESULTS
3. SUPPLEMENTARY TABLES
4. SUPPLEMENTARY FIGURES
5. SUPPLEMENTARY REFERENCES

1. SUPPLEMENTARY METHODS

Data Acquisition

Brain MRI, including Diffusion-weighted images and ^{1}H-MRS were acquired using a single-shot echo-planar imaging (EPI) sequence on a 3 Tesla Siemens Trio MRI scanner (Siemens Healthineers, Erlangen, Germany). The acquisition protocol included 30 diffusion directions with a b-value of 1000 s/mm^2. Imaging parameters were as follows: repetition time (TR) = 8300–8800 ms, echo time (TE) = 84 ms, flip angle = 90°–180°, matrix size = 128 × 128, field of view = 25.6 cm, slice thickness = 2 mm, and isotropic voxel resolution of 2.0 × 2.0 × 2.0 mm^3. Sixty-four axial slices were acquired with anterior-to-posterior phase encoding, and GRAPPA parallel imaging was used with an acceleration factor of 2. The T1 images were used to guide ^{1}H-MRS voxel positioning in the right hippocampus following same protocol as previously published (1).

Participants were scanned using the Mescher-Garwood point-resolved spectroscopy (MEGA-PRESS) Siemens prototype sequence (2) for the measurement of GABA with macromolecules and homocarnosine (GABA+) at 3.0 ppm with the following parameters: repetition time/echo time 1500/68 ms; spectral width: 2000 Hz; editing pulse offset (ON/OFF) 1.9/7.5 ppm; bandwidth of

the editing pulse specified on the scanner 60 Hz; 2048 data points; and voxel sizes and number of transients 15 × 35 × 40 mm^3 and 196 for ACC, 20 × 30 × 40 mm^3 and 300 for right hippocampus, and 30 × 30 × 30 mm^3 and 240 for STC. Before every MRS acquisition, we performed shimming using the vendor-specific shim tool. We further optimized the shim values by manually adjusting the linear shim terms. Finally, unsuppressed water acquisitions were performed with the same region-specific parameters, except 16 transients for concentration quantification. All participants were scanned using the same protocol across sessions. Whenever possible, foam padding was used to minimize head motion, and participants were instructed to remain still during the acquisition.

Diffusion Weighted-Imaging Pre-Processing

Diffusion-weighted imaging (DWI) data were preprocessed using a standardized pipeline based on MRtrix3 and FSL (v6.0.7.12), following established practices in ALPS-based studies (3) (Taoka et al., 2017). Preprocessing steps included denoising, removal of Gibbs ringing artefacts, correction for motion and eddy current distortions, bias field correction, and brain masking. Diffusion tensors were then fitted using FSL's dtifit, and scalar maps including fractional anisotropy (FA) and mean diffusivity (MD) were computed. FA maps were reoriented to the JHU-ICBM-FA-1mm template using a 6-degree-of-freedom transformation to ensure that the Dxx, Dyy, and Dzz components were aligned with respect to the X, Y, and Z axes, thereby accounting for potential head movement effects that could influence ROI placement. All ALPS index computations were performed in native diffusion space. All preprocessed scans underwent thorough visual quality control to exclude motion artefacts, misalignment, and signal dropout. Details of the preprocessing code and parameters are available on demand.

ALPS Index Computation

Following the main preprocessing steps, tensor-derived diffusion metrics were processed using a custom FSL-based pipeline (v6.0.7.12) to extract directional components required for ALPS index computation. In this study, we employed two complementary ROI placement strategies: (1) a manual method, performed in native diffusion space (2) an atlas-based automated registration method to enable reproducible ROI placement adapted to individual anatomy.

For each subject, the three diagonal elements of the diffusion tensor (Dxx, Dyy, Dzz) were isolated using FSL's fslroi, enabling direction-specific analysis of water diffusivity along the right–left (x), anterior–posterior (y), and inferior–superior (z) axes. These components were later used to compute the ALPS index based on established methodology. Additionally, a directional fractional anisotropy (FA) colormap was generated for each scan by multiplying the principal eigenvector (V1) map by the corresponding FA map using FSL's fslmaths. This visualization facilitated quality control and enabled the assessment of local diffusion orientation patterns.

The ALPS index was computed following the methodology described by Taoka et al. (3–5).

Manual ROI placement method

Spherical regions of interest (ROIs) with a diameter of 4 mm were manually placed bilaterally at the level of the lateral ventricles. ROIs were placed in white matter regions corresponding to projection and association fibers, whose predominant orientations (z- and y-axes, respectively) were approximated based on local diffusion directions within native space as shown in **Figure 1a**. Placement was performed using FSLeyes, guided by directionally encoded color (DEC) maps and the tensor component images (Dxx, Dyy, Dzz) to ensure anatomical precision. ROI placement was performed blinded to subject identity and scan timepoint to minimize operator bias.

ROIs were carefully positioned adjacent to medullary veins, which course predominantly along the right–left (x-axis) direction in periventricular regions and are thought to align with perivascular spaces. Operators were blinded to both subject identity and scan timepoint to avoid bias in longitudinal sessions. To enhance measurement specificity, regions where association fibers intersected with subcortical tracts oriented in the x-direction were systematically avoided, in accordance with published recommendations (5).

From each ROI, directional diffusivity values were extracted from the tensor component maps. The ALPS index was then computed using the formula:

ALPS index = mean(Dx_proj, Dx_assoc) / mean(Dy_proj, Dz_assoc)

In this equation, the numerator captures diffusivity along the x-axis (presumed to follow perivascular spaces), while the denominator reflects diffusion orthogonal to PVSs, within structured white matter tracts. A higher ALPS index suggests greater fluid mobility along perivascular pathways and is interpreted as an indicator of more efficient glymphatic function.

The ALPS index was calculated separately for the left and right hemispheres in each scan. A bilateral mean was derived by averaging the two hemispheric values.

Automated ROI Placement method as replication

To ensure reproducible and anatomically consistent ROI placement across participants, an automated pipeline was developed to project standard-space ROIs into individual diffusion space. Four bilateral ROIs, targeting the superior corona radiata (SCR) and superior longitudinal fasciculus (SLF), were defined in MNI space using the JHU-ICBM-FA-2mm atlas. Non-linear registration was then used to map these ROIs to each subject's native space for subject-specific

ALPS index computation. A visual overview of the ROI placement in MNI and subject space is shown in **Supplementary Figures 1b and 1c**, respectively.

More specifically, we used a symmetric diffeomorphic registration approach via ANTs. First, the MNI FA template (JHU-ICBM-FA-2mm) was aligned to each subject's native FA map using antsRegistrationSyNQuick.sh with the -t s option. The resulting affine (0GenericAffine.mat) and nonlinear warp (1Warp.nii.gz) transforms were applied to each ROI using antsApplyTransforms, producing individualized ROIs in diffusion space. ROIs were placed bilaterally in the superior corona radiata (SCR) and superior longitudinal fasciculus (SLF), based on established anatomical definitions. After transformation, diffusivity values were extracted from the Dxx, Dyy, and Dzz maps within each ROI. The ALPS index was computed per hemisphere using the formula provided in the main text.

Control Analysis for Callosal Crossing Fibres

To rule out the possibility that ALPS differences reflected group-dependent variation in callosal diffusivity, we extracted mean Dxx values from an ROI placed in the body of the corpus callosum at the same axial level as the ALPS ROIs (**Supplementary Figure 3a**). This ROI was defined from the JHU white-matter atlas and transformed into each participant's native diffusion space using the same affine mapping applied to the ALPS ROIs. Mean Dxx values were then submitted to the same mixed-effects model used for the ALPS analyses.

Supplementary Methods: Statistical Analysis Details

Statistical analyses were designed to account for the nested structure of the longitudinal dataset, which included repeated DTI measurements acquired across development in both individuals with

22q11DS and HCs. As described above, a total of 258 scans were retained. All scans passed both quality control and preprocessing steps and were included in the final analysis. The distribution of longitudinal scans per participant and inter-scan intervals is reported in **Supplementary Table 2**.

ALPS indices were computed separately for the left and right hemispheres, with outliers removed using interquartile range (IQR ±1.5) filtering applied independently to each side and separately within each group (HC and 22q11DS). Bilateral average ALPS values were then calculated using only scans that retained valid indices in both hemispheres. Detailed counts of retained scans after outlier removal are provided for both manual and automated method in **Supplementary Table 3.**

Psychosis status was assessed using the Structured Interview for Psychosis-Risk Syndromes (SIPS). Of the 85 individuals with 22q11DS, 78 had at least one valid SIPS assessment and were therefore included in the symptom-stratified analyses. Seven individuals were excluded due to missing or invalid SIPS data. Among the included participants, 38 met criteria for moderate-to-severe positive psychotic symptoms (SIPS P1–P5 $\geq$ 3) at any timepoint and were classified as psychosis-positive (PPS+), while the remaining 40 were considered psychosis-negative (PPS−). A total of 123 scans from these 78 individuals were retained for the PPS+ vs PPS− analyses (PPS+ = 62 scans, PPS− = 61 scans). As in the main group-level analysis, outliers were removed using the interquartile range (IQR ±1.5) method, applied separately for each hemisphere and independently within each subgroup.

All statistical analyses used the ALPS indices as dependent variables. To account for repeated measures and inter-individual developmental variability, linear mixed-effects models were implemented. These models included fixed effects for age, sex, and diagnostic group, and a random slope for age at the subject level to capture within-subject variability over time. To assess developmental trajectories, we incorporated interaction terms between age and diagnostic group.

A linear age term was employed for both the 22q11DS vs. HC and PPS+ vs. PPS− comparisons, providing a consistent and interpretable framework for group-by-age interaction effects. The decision to use a linear specification was based on preliminary analyses, which showed no strong evidence for nonlinear age effects and favored model stability. For interpretability, age was mean-centered prior to analysis. This centering approach allows the main group effect to reflect differences in the ALPS index at the average age of the sample, rather than at an arbitrary zero point outside the observed age range. The final model thus included fixed effects for mean-centered age, diagnostic group (coded as 0 = control or PPS−, 1 = 22q11DS or PPS+), their interaction, and sex, with a subject-level random slope for age to account for repeated measurements.

The same statistical modelling approach was applied to ALPS indices derived from both the manual and automated ROI methods, ensuring consistency in the evaluation of developmental trajectories and group effects.

All statistical analyses were conducted in Python (using `statsmodels` and `pingouin`), and all models used fixed effects for group, age, and sex, with subject ID modelled as a random intercept and random slope to account for repeated measures. Statistical significance was set at $p < 0.05$.

MRS Processing and Analysis

All spectral data were processed using Gannet (version 3.1.5) (6). After frequency and phase correction of transients, data were combined to generate GABA-edited spectra. The GABA+, glutamate+glutamine (Glx), and unsuppressed water signals were modeled to calculate GABA and Glx concentrations in international units. T1-weighted images were segmented using SPM12 (7) to calculate gray matter, white matter, and cerebrospinal fluid voxel tissue fractions in the ^{1}H-MRS

voxels. Subsequently, metabolite concentrations were corrected for partial volume effects using the tissue fractions and relaxation effects with the Gasparovic method (8). Data quality was assessed using the creatine signal at 3 ppm to estimate main magnetic field (B_0) drift (before frequency and phase correction) and using the unsuppressed water signal linewidth at full width at half maximum. Software that uses linear combination modeling determines Cramér-Rao lower bounds to express the uncertainty of the fitted signal amplitude. However, Gannet does not provide Cramér-Rao lower bounds but determines fitting quality (called fit error) through the standard deviation of fitting residuals normalized to the height of the modeled peak. The GABA and Glx fit errors generated by Gannet were used for assessing modeling errors in every region. As in previous papers (1,9,10), data with fit errors exceeding 2 standard deviations above the mean of all the data (15%) were excluded from further analyses.

The Glx/GABA ratio was computed and served as a proxy for excitatory/inhibitory (E/I) balance as performed in previous studies (11).

In a subset of 39 participants with 22q11DS (25 males, 14 females; mean age = 21.6 ± 6.9 years), both high-quality spectroscopy and diffusion data were available.

Linear regression models tested the association between ALPS index average (predictor) and Glx/GABA ratio (outcome), controlling for age and sex. Additional regressions using left and right ALPS values separately were performed to assess hemispheric specificity.

Outlier removal was performed using the interquartile range (IQR ±1.5) method, consistent with the statistical procedures applied throughout the study. Outliers were assessed independently for each ALPS index (left and right) as well as for the Glx/GABA ratio. The mean ALPS index was computed only when both left and right values were within non-outlier bounds. This procedure resulted in the exclusion of a single scan based on a low right ALPS index. No outliers were

identified for the Glx/GABA ratio. Consequently, 38 scans were retained for the analyses involving ALPS right and ALPS average, while all 39 scans were included in the model using ALPS left. All data were from unique individuals (i.e., no longitudinal scans), and all models included age and sex as covariates.

FDR correction.

Because key effects (group differences, PPS × age interactions, and ALPS–Glx/GABA associations) were tested across the three ALPS indices (average, left, right), each set of related tests was treated as a family of three comparisons. Raw *p*-values were adjusted using the Benjamini–Hochberg false discovery rate (FDR) procedure. All adjusted *p*-values are summarized in **Supplementary Table 9**.

Cognitive sensitivity analyses

To evaluate whether cognitive functioning influenced glymphatic-related measurements, we performed additional linear mixed-effects models restricted to the 22q11DS cohort, for whom Full-Scale IQ (FSIQ_SC) was available. Because IQ was not collected for HCs, these analyses were conducted within the 22q11DS group only. As these were sensitivity analyses aimed at testing whether IQ moderated PPS-related differences or age-dependent ALPS trajectories, rather than re-estimating full developmental slopes, all models were specified with a subject-level random intercept to account for repeated measures. First, we estimated Age-focused models for each ALPS index (ALPS_avg, ALPS_left, ALPS_right), including fixed effects for PPS status, mean-centred age (Age_c), sex, and mean-centred IQ (FSIQ_SC_c), as well as the PPS × Age_c interaction: Model: ALPS ~ PPS × Age_c + Sex + FSIQ_SC_c + (1 | Subject_ID). Second, we fitted an IQ-

focused moderation model for ALPS_avg to test whether IQ modified PPS-related differences: Model: ALPS_avg ~ PPS × FSIQ_SC_c + Sex + Age_c + (1 | Subject_ID). Finally, to evaluate whether IQ influenced developmental ALPS trajectories independently of PPS status, we fitted an Age × IQ model including fixed effects for Age_c, FSIQ_SC_c, their interaction, sex, and PPS status: Model: ALPS_avg ~ Age_c × FSIQ_SC_c + Sex + PPS + (1 | Subject_ID). Full model specifications and numerical results are reported in the Supplementary Methods, Supplementary Results, and Supplementary Tables 8a–e.

Family Structure (Siblings) Sensitivity Analysis

Because a subset of HCc were siblings of individuals with 22q11.2DS, we evaluated whether family membership introduced non-independence in the data. For this sensitivity analysis, all primary mixed-effects models were re-estimated including an additional family-level random intercept (Family_ID). These models included a subject-level random intercept (1 | Subject_ID), but not the random age slope present in the main developmental models, in order to ensure model convergence and because the goal of this analysis was specifically to test whether family clustering improved model fit. Models with and without the family-level random intercept were compared using likelihood-ratio tests (LRTs) to determine whether accounting for family membership improved model fit. Full LRT statistics and fixed-effect estimates are reported in **Supplementary Table 10**.

2. SUPPLEMENTARY RESULTS

ALPS Index Comparison between HC and 22q11DS – Automated Method

To rule out potential bias arising from manual ROI placement, we re-analysed the data using an automated pipeline for ALPS index computation, as detailed in the *Methods* section. This approach algorithmically defined ROIs adjacent to the lateral ventricles in each subject's native space. We then fitted linear mixed-effects models for the Left, Right, and Average ALPS indices, including Group (HC vs. 22q11DS), Age (mean-centred), and Sex as fixed effects, and allowing for a random slope for Age nested within Subject ID to account for within-subject longitudinal variability. A significant main effect of Group was observed for both the Right ALPS index (p = 0.008; **Supplementary Figure 2c**) and the Average ALPS index (p = 0.003; **Supplementary Figure 2a**), reflecting lower ALPS values in individuals with 22q11DS relative to HCs. The Left ALPS index exhibited a non-significant trend in the same direction (p = 0.129; **Supplementary Figure 2b**). These findings closely replicated the results obtained using manually defined ROIs, supporting the reliability of ALPS index measurements and reinforcing the robustness of the group differences. Full model results are reported in **Supplementary Table 5.**

Supplementary Results - Corpus Callosum Control ROI for Dxx

As a negative-control analysis, we examined whether group differences in diffusivity along the x-axis were present in a bilateral corpus callosum ROI located at the same axial level as the ALPS ROIs (see **Supplementary Figure 3a**). Mixed-effects modelling revealed a significant increase in callosal Dxx in individuals with 22q11.2DS compared with controls ($\beta = 4.82 \times 10^{-4}$, 95% CI [$3.52 \times 10^{-4}$, 6.12×10^{-4}], $p = 1.27 \times 10^{-11}$), see **Supplementary Figure 3b**. This pattern

is opposite to that observed in the ALPS ROIs and therefore indicates that group differences in ALPS are not attributable to crossing callosal fibers.

Supplementary Results - Cognitive sensitivity analyses

- Age-focused models (ALPS ~ PPS × Age + Sex + IQ)

In age-focused models including PPS status, mean-centred age (Age_c), sex, and mean-centred FSIQ_SC (FSIQ_SC_c), we first examined ALPS_avg. PPS status by age showed a significant interaction (PPS_subjectPPS+ × Age_c: $\beta = -0.0159$, SE = 0.0057, $p = 0.006$), indicating that developmental trajectories of ALPS_avg differed between PPS+ and PPS− individuals. Age_c showed a positive main effect ($\beta = 0.0098$, SE = 0.0044, $p = 0.028$), whereas the main effect of PPS status at the mean age and IQ was not significant ($\beta = 0.0497$, SE = 0.0359, $p = 0.17$). Importantly, FSIQ_SC_c did not contribute significantly to ALPS_avg ($\beta = 0.00048$, SE = 0.00153, $p = 0.76$).

For ALPS_index_Left, no fixed-effect term reached significance, and FSIQ_SC_c again showed no association with ALPS ($\beta = -0.00019$, SE = 0.00185, $p = 0.92$). For ALPS_index_Right, PPS status and its interaction with age were robustly associated with ALPS (PPS_subjectPPS+: $\beta = 0.0914$, SE = 0.0383, $p = 0.020$; PPS_subjectPPS+ × Age_c: $\beta = -0.0224$, SE = 0.0062, $p < 0.001$), and Age_c showed a positive effect ($\beta = 0.0164$, SE = 0.0048, $p = 0.0009$). In contrast, FSIQ_SC_c was not significantly related to ALPS_index_Right ($\beta = 0.00155$, SE = 0.00164, $p = 0.35$).

- IQ-focused model (ALPS_avg ~ PPS × IQ + Sex + Age)

In the IQ-focused model for ALPS_avg, which included a PPS_subject × FSIQ_SC_c interaction, the main effect of FSIQ_SC_c remained small and non-significant ($\beta = -0.00251$, SE = 0.00226, $p = 0.27$). The PPS_subjectPPS+ × FSIQ_SC_c interaction term did not reach significance ($\beta = 0.00480$, SE = 0.00318, $p = 0.14$), providing no evidence that IQ moderates the association between PPS status and ALPS_avg. Sex and Age_c covariates were not significant in this model.

Overall, these exploratory analyses indicate that: (i) PPS-related differences and age-dependent trajectories in ALPS, particularly for the right hemisphere and the bilateral average, remain present after adjustment for IQ; and (ii) Full-Scale IQ does not exert a robust main effect on ALPS, nor does it meaningfully moderate PPS-related ALPS differences.

- Age × IQ moderation model (ALPS_avg ~ Age × IQ + PPS + Sex)

To evaluate whether IQ moderated age-related changes in ALPS, we fitted a mixed-effects model including an Age_c × FSIQ_SC_c interaction term, while adjusting for PPS status and sex (as in Supplementary Table 8e). Neither the main effect of FSIQ_SC_c ($\beta = -4.8 \times 10^{-5}$, SE = 0.0016, $p = 0.98$) nor the Age_c × FSIQ_SC_c interaction ($\beta = -2.3 \times 10^{-4}$, SE = 2.47×10^{-4}, $p = 0.34$) reached significance, providing no evidence that cognitive functioning modulated developmental trajectories of ALPS_avg within the 22q11DS group. As expected, PPS status remained a significant predictor in this adjusted model ($\beta = 0.0835$, SE = 0.0341, $p = 0.0155$), confirming that PPS-related differences in ALPS_avg were not explained by IQ or Age × IQ interactions. Full fixed-effect estimates are reported in Supplementary Table 8e.

Supplementary Analysis: Family Structure (Sibling) Effects

To assess whether the presence of siblings among HCs introduced non-independence, we repeated the primary mixed-effects models including a family-level random intercept (Family_ID). Family-level variance was near zero for all ALPS indices. Likelihood-ratio tests comparing models with and without the family random intercept showed no evidence that family membership improved model fit (ALPS_left: $p = 1.00$; ALPS_right: $p = 0.44$; ALPS_avg: $p = 0.90$). These results confirm that sibling membership does not introduce measurable non-independence and therefore does not confound the group-level differences reported in the main text. Full fixed-effect estimates and LRT statistics are reported in **Supplementary Table 10**.

3. SUPPLEMENTARY TABLES

An overview of FDR-corrected *p*-values for all ALPS analyses is presented in Supplementary Table 9.

Supplementary Table 1. Overview of Sample Characteristics and Scan Distribution.

Group	N Participants	N Scans	Mean Age (± SD)	Sex (M/F)
22q11DS	85	143	18.92 ± 6.36	43 / 42
Healthy Controls (HC)	83	115	16.28 ± 6.42	40 / 43
**Total**	**168**	**258**	—	—

Abbreviations: 22q11DS, 22q11.2 deletion syndrome; HC, healthy controls; SD, standard deviation; M/F, male/female.

Supplementary Table 2. Retained ALPS Index Values After Outlier Removal for Manual and Automated Calculation Methods. Values reflect the number of scans retained for each index (Left, Right, Avg) after removing outliers. Average values were retained only if the scan passed outlier thresholds on both the left and right indices.

Method	N Before Outlier Removal	Left Retained (No Outliers)	Right Retained (No Outliers)	Avg Retained (No Outliers)
Manual	258	250	256	249
Automated	258	248	253	246

Abbreviations: ALPS, Analysis Along the Perivascular Space; Avg, bilateral average.

Supplementary table 3. SIPS Assessment and Psychosis Stratification (22q11DS only).

**Assessment Detail**	**n Participants**	**N Scan**
With valid SIPS data	78	123
Psychosis-positive (SIPS ≥ 3)	38	62
Psychosis-negative	40	61
Without SIPS (e.g., age < 6)	7	

Abbreviations: SIPS, Structured Interview for Psychosis-Risk Syndromes; 22q11DS, 22q11.2 deletion syndrome; n, number.

Supplementary Table 4. Fixed effects from linear mixed-effects models comparing ALPS index values between 22q11DS individuals and HCs.

Separate models were run for the bilateral average (**Supplementary Table 4a**), left (**Supplementary Table 4b**) and right (**Supplementary Table 4c**) hemisphere indices. Fixed effects included age (mean-centered), group (0 = control, 1 = 22q11DS), sex, and their interaction. Bold *p*-values indicate statistical significance ($p < 0.05$).

Supplementary Table 4a

	avg_ind		
Predictors	*Estimates*	*CI*	*p*
(Intercept)	1.54	1.50 – 1.58	**<0.001**
Age centered	0.00	-0.00 – 0.01	0.126
VCFS Status [1]	-0.05	-0.10 – -0.01	**0.017**
Sex [1]	-0.00	-0.05 – 0.04	0.906
Age centered × VCFS Status [1]	-0.00	-0.01 – 0.00	0.235

Supplementary Table 4b

	ALPS_index_Left		
Predictors	*Estimates*	*CI*	*p*
(Intercept)	1.51	1.47 – 1.56	**<0.001**
Age centered	0.00	-0.00 – 0.01	0.654
VCFS Status [1]	-0.03	-0.08 – 0.02	0.253
Sex [1]	0.01	-0.04 – 0.06	0.630
Age centered × VCFS Status [1]	-0.00	-0.01 – 0.00	0.235

Supplementary Table 4c

	ALPS_index_Right		
Predictors	*Estimates*	*CI*	*p*
(Intercept)	1.56	1.51 – 1.61	**<0.001**
Age centered	0.01	0.00 – 0.01	**0.037**
VCFS Status [1]	-0.07	-0.13 – -0.01	**0.022**
Sex [1]	-0.00	-0.06 – 0.06	0.890
Age centered × VCFS Status [1]	-0.00	-0.01 – 0.01	0.508

Abbreviations: ALPS, Analysis Along the Perivascular Space; CI, confidence interval; *p*, *p*-value; VCFS, velo-cardio-facial syndrome (22q11.2 deletion syndrome); Sex [1], dummy-coded sex variable (1 = male, 0 = female); Status [1], dummy-coded VCFS status variable; Age centered, age mean-centered prior to model fitting.

Supplementary Table 5. Fixed effects from linear mixed-effects models comparing ALPS index values between 22q11DS individuals and HCs using the automated ROI method.

Supplementary Table 5a: ALPS Index automated - Average

Fixed Effects Coefficients:

Term	Coef.	Std.Err.	z	p	[0.025, 0.975]
Intercept	1.441	0.016	90.62	0.000	[1.410, 1.472]
Sex[T.Male]	0.023	0.018	1.27	0.202	[-0.012, 0.057]
VCFS_Status	-0.053	0.018	-2.92	**0.003**	[-0.089, -0.017]
Age_centered	-0.002	0.002	-1.10	0.271	[-0.006, 0.002]
VCFS_Status x Age_centered	-0.001	0.003	-0.49	0.622	[-0.007, 0.004]

Supplementary Table 5b: ALPS Index automated - Left

Fixed Effects Coefficients:

Term	Coef.	Std.Err.	z	p	[0.025, 0.975]
Intercept	1.449	0.021	69.60	0.000	[1.409, 1.490]
Sex[T.Male]	0.065	0.020	3.26	**0.001**	[0.026, 0.104]
VCFS_Status	-0.031	0.021	-1.52	0.129	[-0.072, 0.009]
Age_centered	0.020	160438.923	0.00	1.000	[-314454.491, 314454.531]
VCFS_Status x Age_centered	0.013	223018.680	0.00	1.000	[-437108.567, 437108.593]

Supplementary Table 5c: ALPS Index automated - Right:

Fixed Effects Coefficients:

Term	Coef.	Std.Err.	z	p	[0.025, 0.975]
Intercept	1.436	0.024	59.68	0.000	[1.389, 1.483]
Sex[T.Male]	-0.025	0.023	-1.09	0.276	[-0.069, 0.020]
VCFS_Status	-0.063	0.024	-2.64	**0.008**	[-0.110, -0.016]
Age_centered	0.065	179175.052	0.00	1.000	[-351176.584, 351176.714]
VCFS_Status x Age_centered	-0.013	nan	nan	nan	[nan, nan]

Abbreviations: ALPS, Analysis Along the Perivascular Space; Coef., coefficient; Std.Err., standard error; CI, confidence interval; p, p-value; z, z-statistic; VCFS, velo-cardio-facial

syndrome (22q11.2 deletion syndrome); Sex[T.Male], dummy-coded sex variable (1 = male, 0 = female); Age_centered, age mean-centered prior to modelling; VCFS Status × Age_centered, interaction term; nan, not-a-number (non-estimable model parameter).

Supplementary Table 6. Fixed effects from linear mixed-effects models predicting ALPS index values as a function of age, group (0 = PPS−, 1 = PPS+), sex, and their interaction. Separate models were fitted for the bilateral average, right, and left hemisphere indices. Age was mean-centered. Bold *p*-values indicate statistical significance ($p < 0.05$).

Supplementary Table 6a

	ALPS_index_Avg		
Predictors	*Estimates*	*CI*	*p*
(Intercept)	1.45	1.40 – 1.51	**<0.001**
Age centered	0.01	0.00 – 0.02	**0.039**
Group [1]	0.05	-0.02 – 0.11	0.141
Sex [1]	0.01	-0.05 – 0.07	0.805
Age centered × Group [1]	-0.01	-0.02 – -0.00	**0.009**

Supplementary Table 6b

	ALPS_index_Left		
Predictors	*Estimates*	*CI*	*p*
(Intercept)	1.48	1.42 – 1.54	**<0.001**
Age centered	0.00	-0.01 – 0.01	0.785
Group [1]	0.00	-0.07 – 0.07	0.955
Sex [1]	-0.00	-0.07 – 0.07	0.987
Age centered × Group [1]	-0.01	-0.02 – 0.01	0.292

Supplementary Table 6c

	ALPS_index_Right		
Predictors	*Estimates*	*CI*	*p*
(Intercept)	1.44	1.37 – 1.51	**<0.001**
Age centered	0.02	0.01 – 0.03	**0.001**
Group [1]	0.09	0.01 – 0.17	**0.025**
Sex [1]	0.03	-0.05 – 0.11	0.504
Age centered × Group [1]	-0.02	-0.03 – -0.01	**0.001**

Abbreviations: ALPS, Analysis Along the Perivascular Space; CI, confidence interval; p, p-value; Group [1], dummy-coded psychosis-risk group (1 = PPS+, 0 = PPS−); Sex [1], dummy-coded sex variable (1 = male, 0 = female); Age centered, age mean-centered prior to modelling; Age centered × Group [1], interaction term.

Supplementary table 7: Results from linear regression models testing the association between ALPS index and Glx/GABA ratio.

Each model included one ALPS index (left, right, or average) as the predictor and Glx/GABA ratio (measured in the right hippocampus) as the dependent variable. All models controlled for age and sex. Table reports beta coefficients, *p*-values for each predictor, and the model R^2. Significant associations between ALPS and Glx/GABA ratio were found in all models, indicating a robust inverse relationship between glymphatic function and excitatory/inhibitory imbalance in 22q11DS.

**ALPS Predictor**	β (ALPS)	p (ALPS)	p (age)	p (Sex)	R^2
ALPS_index_Left	-7.662	**0.0181**	0.3338	0.8502	0.159
ALPS_index_Right	-6.765	**0.0148**	0.4562	0.7327	0.174
ALPS_index_Average	-11.015	**0.0022**	0.2482	0.7912	0.255

Abbreviations: ALPS, Analysis Along the Perivascular Space; Glx, glutamate + glutamine; GABA, gamma-aminobutyric acid; β, standardized beta coefficient; p, p-value; R^2, coefficient of determination; 22q11DS, 22q11.2 deletion syndrome.

Supplementary Table 8. Fixed-effect estimates from mixed-effects models testing the contribution of cognitive functioning (FSIQ_SC) to ALPS measures in 22q11.2DS.

Mixed-effects models were fitted including fixed effects for PPS status (subject-level ever-PPS+ vs never-PPS−), mean-centred age (Age_c), sex, and mean-centred Full-Scale IQ (FSIQ_SC_c). Subject was included as a random intercept. "Age-focused" models evaluated ALPS_avg, ALPS_left, and ALPS_right as outcomes with PPS × Age_c interaction. An additional "IQ-focused" model tested PPS × FSIQ_SC_c on ALPS_avg. Bold *p*-values indicate statistical significance at $p < 0.05$. ALPS units are dimensionless indices.

Supplementary table 8a – ALPS average/Age

Term	Estimate	Std. Error	df	t value	p value
(Intercept)	1.466	0.0317	86.46	46.208	<0.0001
**PPS_subjectPPS+**	0.04971	0.03589	77.04	1.385	0.1700
**Age_c**	0.00982	0.00442	124.8	2.222	**0.0281**
SexM	0.01803	0.03647	79.84	0.494	0.6223
FSIQ_SC_c	0.00048	0.00153	92.46	0.313	0.7552
**PPS_subjectPPS+:Age_c**	–0.01594	0.00570	126.6	–2.797	**0.0060**

Supplementary table 8b – ALPS left/Age

Term	Estimate	Std. Error	df	t value	p value
(Intercept)	1.496	0.03813	86.36	39.218	<0.0001
PPS_subjectPPS+	0.00747	0.04295	76.03	0.174	0.8620
Age_c	0.00335	0.00546	126.1	0.613	0.5410
SexM	0.01101	0.04370	78.85	0.252	0.8020
FSIQ_SC_c	–0.00019	0.00185	90.36	–0.102	0.9190
PPS_subjectPPS+:Age_c	–0.01004	0.00702	127.0	–1.430	0.1550

Supplementary table 8c – ALPS right/Age

Term	Estimate	Std. Error	df	t value	p value
(Intercept)	1.436	0.03395	78.42	42.308	<0.0001
**PPS_subjectPPS+**	0.09143	0.03831	67.87	2.387	**0.0198**
**Age_c**	0.01643	0.00480	125.1	3.419	**0.00085**
SexM	0.02525	0.03896	70.82	0.648	0.5190
FSIQ_SC_c	0.00155	0.00164	83.76	0.945	0.3472
**PPS_subjectPPS+:Age_c**	–0.02241	0.00618	126.9	–3.624	**0.00042**

Supplementary table 8d – ALPS average/IQ

Term	Estimate	Std. Error	df	t value	p value
(Intercept)	1.461	0.03280	85.19	44.559	<0.0001
PPS_subjectPPS+	0.05380	0.03697	77.94	1.455	0.1500
FSIQ_SC_c	–0.00251	0.00226	101.4	–1.108	0.2700
SexM	0.02886	0.03802	81.57	0.759	0.4500
Age_c	0.00000	0.00290	126.3	0.000	1.0000
PPS_subjectPPS+:FSIQ_SC_c	0.00480	0.00318	95.00	1.507	0.1350

Supplementary table 8e - ALPS average / Age × IQ

Term	Estimate	Std. Error	df	t value	p value
(Intercept)	1.460	0.0298	93.58	49.03	<0.0001
Age_c	0.00073	0.00262	123.8	0.279	0.7808
FSIQ_SC_c	−0.00005	0.00160	91.08	−0.030	0.9763
SexM	0.0217	0.0385	79.49	0.563	0.5750
PPS_subject	0.0835	0.0340	125.0	2.453	0.0155
Age_c:FSIQ_SC_c	−0.00023	0.00025	122.1	−0.949	0.3447

Across all models, FSIQ_SC_c showed no significant main effect on ALPS measures (all $p > 0.25$), and no significant PPS × IQ interaction was observed ($p = 0.14$ for ALPS_avg). In contrast, PPS status and PPS × Age interactions remained significant, particularly for ALPS_index_Right and ALPS_avg, indicating divergent developmental trajectories between PPS+ and PPS− individuals. These results suggest that ALPS differences are not driven by IQ and are robust to cognitive heterogeneity within the 22q11.2DS cohort.

Abbreviations: ALPS, Analysis Along the Perivascular Space; 22q11.2DS, 22q11.2 deletion syndrome; Std. Error, standard error; df, degrees of freedom; t value, t-statistic; *p*, *p*-value; PPS+, presence of clinically significant positive psychotic symptoms; PPS−, absence of clinically significant positive psychotic symptoms; PPS_subject, subject-level PPS status dummy-coded (1 = PPS+, 0 = PPS−); SexM, dummy-coded sex variable (1 = male, 0 = female); Age_c, age mean-centred prior to modelling; FSIQ_SC_c, standardized Full-Scale IQ mean-centred; PPS × Age_c, interaction term between PPS status and age; PPS × FSIQ_SC_c, interaction term between PPS status and FSIQ.

Supplementary Table 9. False discovery rate (FDR)–adjusted *p*-values for ALPS analyses

Analysis block	ALPS index	Raw p	FDR-adjusted p	Corresponding main table
22q11DS vs HC (Manual ROIs)	Average	0.017	0.033	Supplementary Table 4a
22q11DS vs HC (Manual ROIs)	Right	0.022	0.033	Supplementary Table 4c
22q11DS vs HC (Manual ROIs)	Left	0.253	0.253	Supplementary Table 4b
22q11DS vs HC (Automated ROIs)	Average	0.003	0.009	Supplementary Table 5a
22q11DS vs HC (Automated ROIs)	Right	0.008	0.012	Supplementary Table 5c
22q11DS vs HC (Automated ROIs)	Left	0.129	0.129	Supplementary Table 5b
PPS × Age interaction	Average	0.009	0.009	Supplementary Table 6a
PPS × Age interaction	Right	0.001	0.003	Supplementary Table 6c
PPS × Age interaction	Left	0.292	0.292	Supplementary Table 6b
ALPS – Glx/GABA (MRS)	Average	0.0022	0.0066	Supplementary Table 7
ALPS – Glx/GABA (MRS)	Right	0.0148	0.0181	Supplementary Table 7
ALPS – Glx/GABA (MRS)	Left	0.0181	0.0181	Supplementary Table 7

Supplementary Table 9. False discovery rate (FDR)–adjusted *p*-values for ALPS analyses. FDR correction (Benjamini–Hochberg) was applied separately within each family of ALPS indices (average, left, right) for key effects tested across indices, including: (i) group differences (22q11DS vs HC) for manual and automated ROI methods, (ii) psychosis-risk trajectories (PPS × age interaction), and (iii) ALPS–Glx/GABA associations. The table reports raw and adjusted *p*-values for each test, as referenced in Supplementary Tables 4–7.

Abbreviations: ALPS, Analysis Along the Perivascular Space; 22q11DS, 22q11.2 deletion syndrome; HC, healthy controls; ROI, region of interest; PPS, clinically significant positive

psychotic symptoms; Glx, glutamate + glutamine; GABA, gamma-aminobutyric acid; MRS, magnetic resonance spectroscopy; FDR, false discovery rate; *p*, *p*-value.

Supplementary Table 10. Mixed-effects models including a family-level random intercept (Family_ID). Fixed-effect estimates and likelihood-ratio tests.

Supplementary Table 10a - Fixed effects (models with subject- and family-level random intercepts)

Linear mixed-effects analyses assessing the impact of family structure on ALPS indices. Supplementary table 10a reports fixed-effect estimates from models including random intercepts for subject (to account for repeated measures) and family (Family_ID) to account for potential non-independence introduced by sibling pairs. Supplementary table 10b reports likelihood-ratio tests comparing models with and without the family-level random intercept. Family-level variance was near zero for all ALPS indices, and model fit did not improve with the inclusion of the family random effect (all $p > 0.43$), indicating that sibling membership did not introduce detectable clustering. Importantly, the significant group difference in right-hemisphere ALPS remained unchanged, confirming that family structure is not a confounder in the present dataset.

**Hemisphere**	**Term**	**Estimate**	**Std. Error**	**df**	***p*-value**
**Average**	(Intercept)	1.541	0.022	149	<0.001
Average	age_c	0.003	0.002	231	0.254
Average	vcfs_statusVCFS	-0.042	0.025	176	0.092
Average	sex_statusMale	0.007	0.025	170	0.786
Average	age_c:vcfs_statusVCFS	-0.003	0.003	249	0.283
**Right**	(Intercept)	1.561	0.027	161	<0.001
Right	age_c	0.0057	0.0029	242	0.053
Right	vcfs_statusVCFS	-0.0699	0.0298	166	0.020
Right	sex_statusMale	-0.0034	0.0296	163	0.909

Hemisphere	Term	Estimate	Std. Error	df	*p*-value
Right	age_c:vcfs_statusVCFS	-0.0048	0.0037	248	0.193
**Left**	(Intercept)	1.521	0.0246	201	<0.001
Left	age_c	0.0003	0.0028	251	0.912
Left	vcfs_statusVCFS	-0.0144	0.0281	187	0.608
Left	sex_statusMale	0.0175	0.0278	177	0.529
Left	age_c:vcfs_statusVCFS	-0.0019	0.0036	249	0.609

ALPS indices were modelled using linear mixed-effects models with fixed effects of age (centered), group (22q11.2DS vs HC), sex (Male vs Female), and the Age × Group interaction, and random intercepts for subject and family (Family_ID). df = Satterthwaite degrees of freedom.

Supplementary Table 10b - Likelihood-ratio tests for family-level random effect

Hemisphere	Model comparison	AIC (no family)	AIC (with family)	χ^2	df	*p*-value
ALPS_left	(1 | subj) vs (1 | subj) + (1 | family_id)	-69.88	-67.88	0.000	1	1.000
ALPS_right	(1 | subj) vs (1 | subj) + (1 | family_id)	-71.32	-69.92	0.599	1	0.439
ALPS_avg	(1 | subj) vs (1 | subj) + (1 | family_id)	-166.62	-164.64	0.015	1	0.904

Likelihood-ratio tests compared models with and without a family-level random intercept (Family_ID). For all ALPS indices, adding the family-level random effect did not improve model fit (all $p > 0.43$), indicating absence of detectable sibling-level clustering in ALPS values.

Abbreviations: ALPS, Analysis Along the Perivascular Space; 22q11.2DS, 22q11.2 deletion syndrome; HC, healthy controls; VCFS, velo-cardio-facial syndrome; Std. Error, standard error; df, degrees of freedom; p, p-value; age_c, age mean-centered prior to modelling;

vcfs_statusVCFS, dummy-coded VCFS status variable (1 = 22q11.2DS, 0 = HC); sex_statusMale, dummy-coded sex variable (1 = male, 0 = female); age_c × vcfs_statusVCFS, interaction term; AIC, Akaike Information Criterion; χ^2, chi-square statistic; Family_ID, family-level identifier; (1 | subj), random intercept for subject; (1 | family_id), random intercept for family.

4. SUPPLEMENTARY FIGURES

Supplementary Figure 1. ALPS Index Calculation

(a) Manual ROI Placement Approach

Schematic representation of the manual method for calculating the ALPS index. ROIs were manually placed in projection and association fiber areas adjacent to the lateral ventricles, guided by colored FA maps.

(b) Automated Method: ROI Placement on MNI template: JHU-ICBM-FA-2mm atlas with 4 ROI templates: Yellow: Left and Right projection ROIs, Blue: Left and Right association ROIs

(c) Automated Method: ROIs after transforming into subject space (randomly picked subject): Subject specific colormap obtained from the FA map plus 4 ROIs warped: Yellow: Left and Right projection ROIs, Blue: Left and Right association ROIs.

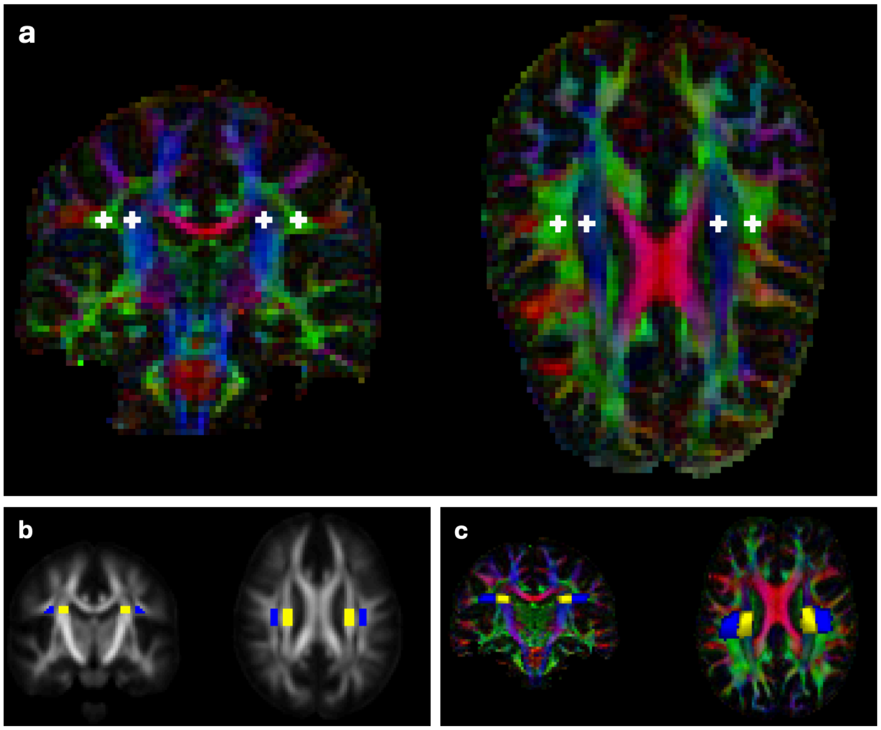

Supplementary Figure 2. Age-related trajectories of ALPS index based on automated ROI placement.

(A) Average ALPS index plotted against age in individuals with 22q11.2 deletion syndrome (22q11DS; red) and healthy controls (HC; blue), using values derived from the automated ROI extraction pipeline. Each dot represents a scan; dashed lines connect longitudinal scans from the same subject. Shaded areas represent 95% confidence intervals of the fitted linear mixed-effects models. A significant group difference was observed, with lower average ALPS values in the 22q11DS group ($p = 0.003$).

(B) Left hemisphere ALPS index showed a non-significant trend toward reduced values in 22q11DS compared to HC ($p = 0.129$). Sex was a significant predictor, with higher ALPS index in males compared to females ($p = 0.001$).

(C) Right hemisphere ALPS index revealed a significant group difference, with lower values in the 22q11DS group ($p = 0.008$).

All models included group, age (mean-centered), sex, and subject-level random slopes for age to account for repeated measures. Black asterisks indicate significant group differences, and grey asterisks indicate significant age effects. *$p < 0.05$, **$p < 0.01$, ***$p < 0.001$, ****$p < 0.0001$; ns = not significant.

Abbreviations: ALPS, Analysis Along the Perivascular Space; HC, healthy controls; VCFS, velo-cardio-facial syndrome (22q11.2 deletion syndrome); *p*, *p*-value.

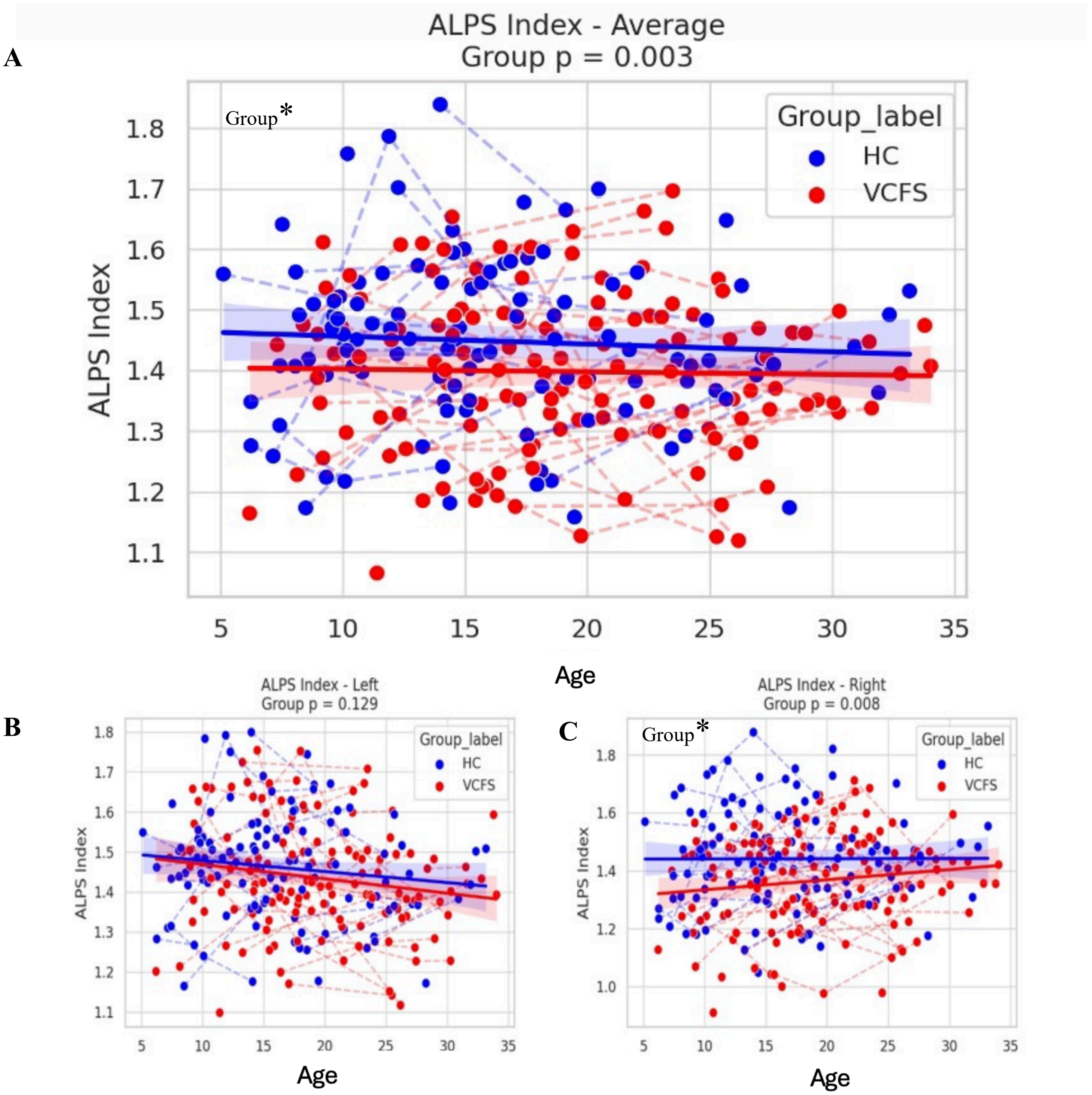
A
ALPS Index - Average
Group p = 0.003
Group*
Group_label
HC
VCFS
ALPS Index
Age
B
ALPS Index - Left
Group p = 0.129
Group_label
HC
VCFS
ALPS Index
Age
C
ALPS Index - Right
Group p = 0.008
Group*
Group_label
HC
VCFS
ALPS Index
Age

Supplementary Figure 3a - Corpus Callosum placement left and right ROIs (in yellow) in MNI space and in alignment with ALPS ROI placement (in blue).

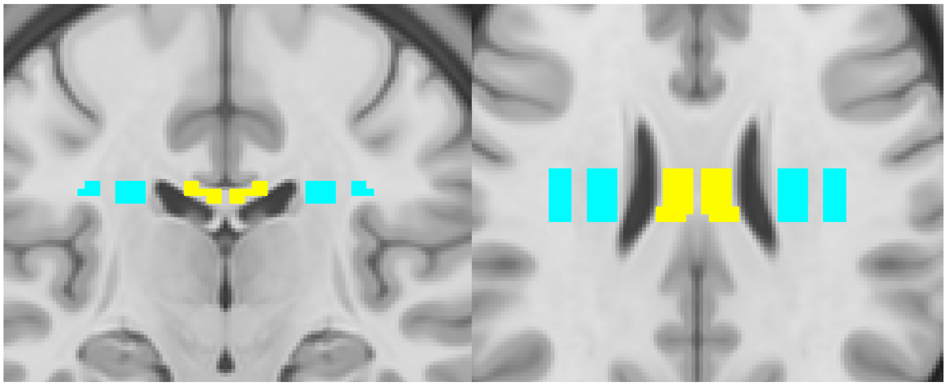

Supplementary Figure 3b: Group difference in age-related trajectories of corpus callosum Dxx fibers in Controls and 22q11DS

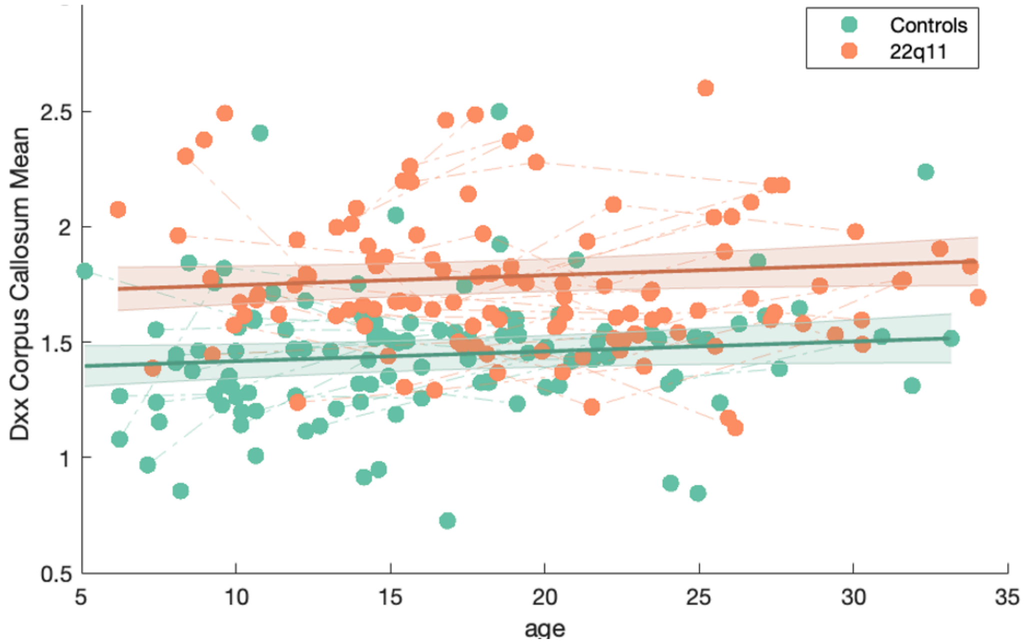

Abbreviations: Dxx, diffusivity along the x-axis; Controls, healthy controls; 22q11.2 deletion syndrome.

5. SUPPLEMENTARY REFERENCES

1. Mancini V, Saleh MG, Delavari F, Bagautdinova J, Eliez S. Excitatory/Inhibitory Imbalance Underlies Hippocampal Atrophy in Individuals With 22q11.2 Deletion Syndrome With Psychotic Symptoms. Biol Psychiatry. 2023 Oct 1;94(7):569–79.

2. Saleh MG, Rimbault D, Mikkelsen M, Oeltzschner G, Wang AM, Jiang D, et al. Multi-vendor standardized sequence for edited magnetic resonance spectroscopy. NeuroImage. 2019 Apr 1;189:425–31.

3. Taoka T, Masutani Y, Kawai H, Nakane T, Matsuoka K, Yasuno F, et al. Evaluation of glymphatic system activity with the diffusion MR technique: diffusion tensor image analysis along the perivascular space (DTI-ALPS) in Alzheimer's disease cases. Jpn J Radiol. 2017 Apr;35(4):172–8.

4. Taoka T, Ito R, Nakamichi R, Nakane T, Sakai M, Ichikawa K, et al. Diffusion-weighted image analysis along the perivascular space (DWI–ALPS) for evaluating interstitial fluid status: age dependence in normal subjects. Jpn J Radiol. 2022 Sep 1;40(9):894–902.

5. Taoka T, Ito R, Nakamichi R, Nakane T, Kawai H, Naganawa S. Diffusion Tensor Image Analysis ALong the Perivascular Space (DTI-ALPS): Revisiting the Meaning and Significance of the Method. Magn Reson Med Sci. 2024 Apr 2;23(3):268–90.

6. Edden RAE, Puts NAJ, Harris AD, Barker PB, Evans CJ. Gannet: A batch-processing tool for the quantitative analysis of gamma-aminobutyric acid–edited MR spectroscopy spectra. J Magn Reson Imaging JMRI. 2014 Dec;40(6):1445–52.

7. Ashburner J, Friston KJ. Unified segmentation. NeuroImage. 2005 Jul 1;26(3):839–51.

8. Gasparovic C, Song T, Devier D, Bockholt HJ, Caprihan A, Mullins PG, et al. Use of tissue water as a concentration reference for proton spectroscopic imaging. Magn Reson Med. 2006 Jun;55(6):1219–26.

9. Wenneberg C, Nordentoft M, Rostrup E, Glenthøj LB, Bojesen KB, Fagerlund B, et al. Cerebral Glutamate and Gamma-Aminobutyric Acid Levels in Individuals at Ultra-high Risk for Psychosis and the Association With Clinical Symptoms and Cognition. Biol Psychiatry Cogn Neurosci Neuroimaging. 2020 Jun;5(6):569–79.

10. Saleh MG, Papantoni A, Mikkelsen M, Hui SCN, Oeltzschner G, Puts NA, et al. Effect of Age on GABA+ and Glutathione in a Pediatric Sample. AJNR Am J Neuroradiol. 2020 Jun;41(6):1099–104.

11. Conen S, Gregory CJ, Hinz R, Smallman R, Corsi-Zuelli F, Deakin B, et al. Neuroinflammation as measured by positron emission tomography in patients with recent onset and established schizophrenia: implications for immune pathogenesis. Mol Psychiatry. 2021 Sep;26(9):5398–406.
